# Registered Prevalence and Regional Characteristics of Congenital Anomalies in the Turkistan Region

**DOI:** 10.3390/ijerph23060722

**Published:** 2026-05-28

**Authors:** Ardak Ayazbekov, Ainash Oshibayeva, Secil Ozkan, Gulzhaukhar Taskinova, Makpal Taubekova

**Affiliations:** 1 Faculty of Medicine, Khoja Akhmet Yassawi International Kazakh-Turkish University, Turkestan 161200, Kazakhstan; ardak.ayazbekov@ayu.edu.kz (A.A.); ainash.oshibayeva@ayu.edu.kz (A.O.); gulzhaukhar.taskynova@ayu.edu.kz (G.T.); 2 Department of Public Health, Faculty of Medicine, Gazi University, 06500 Ankara, Turkey; secilozkan70@gmail.com

**Keywords:** congenital anomalies, congenital disorders, Turkistan region, epidemiology, pregnancy, screening, forecasting, regression

## Abstract

**Highlights:**

**Public health relevance—How does this work relate to a public health issue?**

- Congenital anomalies are a major cause of perinatal morbidity and mortality; this study quantifies their prevalence and trends in the Turkistan Region (Kazakhstan) using 2020–2024 regional perinatal data.
- The analysis documents a steady increase in registered congenital anomalies (overall prevalence 1.6%; rising from 0.8% in 2020 to 2.4% in 2024), highlighting the need for strengthened population-level surveillance and prevention.

**Public health significance—Why is this work of significance to public health?**

- The upward trend suggests a growing burden for maternal–child health services and underscores potential gaps in preconception care, prenatal screening coverage, and early detection pathways at the regional level.
- Regional characterization of anomaly patterns (including a high proportion of cardiovascular anomalies) provides evidence to prioritize screening capacity, referral pathways, and perinatal resource planning.

**Public health implications—What are the key implications or messages for practitioners, policy makers and/or researchers in public health?**

- Practitioners and health systems should strengthen preconception counseling (e.g., folic acid use), expand timely first- and second-trimester screening, and improve access to specialist diagnostics (including fetal echocardiography) to support early detection and management.
- Policy makers and researchers should develop/strengthen a regional congenital anomaly registry and use routine data to monitor trends, identify modifiable risk factors, and evaluate the impact of screening and preventive interventions.

**Abstract:**

Background: Congenital disorders (CDs) are a major contributor to perinatal morbidity and mortality, and their prevalence may vary by region. This study aimed to assess the epidemiological characteristics and regional features of congenital anomalies (CAs) in the Turkistan Region and to evaluate temporal trends and forecast near-term dynamics. Methods: A retrospective analysis was conducted using data from regional perinatal centers and medical institutions in the Turkistan Region for 2020–2024. The total number of registered pregnancies and diagnosed CA cases were summarized annually. Results: From 2020 to 2024, 53,169 pregnancies were registered and 848 cases of CAs were identified, yielding an overall prevalence of 1.6%. The registered prevalence of CAs increased from 0.8% in 2020 to 2.4% in 2024. This temporal increase coincided with the expansion of prenatal screening and diagnostic services in the region during the study period. Conclusions: The findings demonstrate an increase in the registered prevalence of CAs in the Turkistan Region over the study period. The observed trend may reflect both changes in case detection and registration practices, as well as possible epidemiological influences, highlighting the importance of continued surveillance and prenatal diagnostic services.

## 1. Introduction

Congenital disorders (CDs) represent one of the most pressing challenges in contemporary biomedical science and public health systems worldwide. Congenital disorders, also referred to as birth defects, are defined by the World Health Organization as structural or functional abnormalities that occur during intrauterine life and can be identified prenatally, at birth, or later in life. These conditions represent a heterogeneous group of disorders that may result from genetic, environmental, or multifactorial causes and include structural malformations, chromosomal abnormalities, and metabolic disorders. Within the broader category of congenital disorders, congenital anomalies (CAs) represent a specific subgroup primarily characterized by structural developmental abnormalities [1]. In the present study, the focus is placed specifically on congenital anomalies (CAs).

According to data from the World Health Organization, approximately 4–6% of newborns globally are diagnosed each year with various congenital disorders. These conditions are not only among the leading causes of neonatal and early childhood mortality but also contribute substantially to reduced quality of life among surviving children, long-term disability, and increased medical and social costs [2]. Consequently, the prevention, early detection, and epidemiological surveillance of congenital anomalies (CAs) are recognized as priority areas in public health policies across many countries.

The scientific literature widely acknowledges the multifactorial etiology of congenital anomalies. In addition to genetic predisposition, socio-economic conditions, environmental exposures, and medical factors play a significant role in their development. In particular, chemical, physical, and biological agents affecting the maternal organism during pregnancy, as well as maternal somatic and infectious diseases, may disrupt normal fetal morphogenesis and increase the risk of congenital anomalies [3]. Although the underlying mechanisms of these influences have not yet been fully elucidated, their clinical and epidemiological significance has been well established.

At the international level, epidemiological surveillance systems such as EUROCAT (European Surveillance of Congenital Anomalies) and the International Clearinghouse for Birth Defects Surveillance and Research (ICBDSR) enable comprehensive assessment of the structure, prevalence, and temporal trends of congenital anomalies [4]. Data derived from these registries demonstrate that the distribution of congenital anomalies is strongly influenced by population ethnic composition, socio-economic conditions, environmental factors, as well as the accessibility and quality of healthcare services.

In the Republic of Kazakhstan, the overall prevalence of congenital anomalies has remained relatively stable over the past decade; however, regional disparities have not been sufficiently investigated. According to national statistical data, an average of 54.1 cases of congenital anomalies are registered per 1000 live births [5], which is considered high compared with international benchmarks. This situation highlights the need to further improve prevention strategies, screening programs, and early diagnostic systems for congenital anomalies in the country.

The Turkistan Region is characterized by distinct socio-demographic and environmental features [6]. High population density, the predominance of rural settlements, uneven development of medical infrastructure, and increasing anthropogenic environmental pressures may contribute to an elevated risk of congenital anomalies in the region. In addition, specific patterns of reproductive behavior, inadequate preconception care, and insufficient intake of folic acid and other essential micronutrients among the population are considered significant risk factors.

Accordingly, the present study aims to provide a comprehensive assessment of the epidemiological characteristics of CAs registered in the Turkistan Region during the period from 2020 to 2024. The relevance of this research is determined by the need to develop effective measures for the prevention and early detection of congenital anomalies, as well as for the planning and improvement of perinatal care, based on robust region-specific evidence.

In Kazakhstan, the number of studies devoted to the epidemiology of CAs remains limited, particularly with regard to systematic and long-term analyses at the regional level. Existing research is largely focused on individual nosological forms, specific medical institutions, or relatively short observation periods. As a result, the available evidence does not allow for a comprehensive assessment of regional patterns, temporal dynamics, and structural characteristics of congenital anomaly prevalence.

Studies conducted in the southern regions of Kazakhstan, including the Turkistan Region, indicate an increasing trend in the registered prevalence of congenital anomalies. These findings suggest the presence of region-specific factors influencing the occurrence of CAs and highlight the need for expanded epidemiological investigations at the regional level.

As part of the present research project, a preliminary phase analysis conducted during 2020–2022 demonstrated that the prevalence of CAs among live-born infants at the Turkistan City Regional Perinatal Center No. 3 was 0.84%, with a 1.8-fold increase observed over the three-year period [7]. The identified upward trend was attributed to the environmental and socio-economic characteristics of the region.

Similar trends have also been reported in the Zhambyl Region. Kemelbekov et al. (2016) found that the prevalence of congenital heart defects increased from 1.09% to 1.26% between 2014 and 2015, with ventricular septal defects and patent ductus arteriosus predominating in the structural distribution [8]. These findings underscore the leading role of cardiovascular anomalies within the overall structure of CAs and their direct impact on neonatal mortality and long-term disability.

National-level data also indicate pronounced interregional differences in the prevalence of congenital anomalies. An analysis of official statistics from Kazakhstan and the East Kazakhstan Region for the period 2007–2012 conducted by Rakhypbekov et al. (2016) demonstrated higher rates of CAs in the Zhambyl, South Kazakhstan, North Kazakhstan, Akmola, and Mangystau regions [9]. It was noted that in some areas these elevated rates may be associated with environmental factors, including radiation exposure.

Numerous studies demonstrate the multifactorial nature of congenital anomaly development. Syssoyev et al. (2024) reported that maternal somatic diseases before and during pregnancy, viral infections, spontaneous miscarriages, and pregnancy complications significantly increase the risk of congenital heart defects [10]. These findings are consistent with the results reported by Aldasheva et al. (2010), who showed that maternal age, a complicated obstetric history, and infectious diseases have a direct impact on the development of CAs [11].

The significance of infectious factors has also been confirmed in a study conducted by Tusupkaliyev et al. (2015) [12]. The authors found that a substantial proportion of newborns with congenital heart defects were diagnosed with cytomegalovirus and mixed infections, with these conditions being confirmed by polymerase chain reaction (PCR) testing.

Studies focused on genetic monitoring indicate that the specific etiology of a substantial proportion of CAs remains unidentified. Assykbaeva et al. (2025) [13] reported that the prevalence of CAs varies across regions and, in some cases, may be associated with folic acid deficiency and environmental factors [14]. Similarly, Mirzarakhimova et al. (2021) characterized a considerable share of CAs as “sporadic,” emphasizing the absence of clearly identifiable causal factors in many cases [15].

Comparable patterns have been observed in international studies. In a study by Hassan Toufaily et al. (2016), more than 70% of congenital anomaly cases were reported to have an unknown etiology [16].

Thus, the available literature highlights the multifactorial etiology of congenital anomalies, their regional variability, and the leading role of cardiovascular defects within their overall structure. However, in Kazakhstan, including the Turkistan Region, there remains a lack of studies providing a comprehensive assessment of the dynamics and structural patterns of CAs over extended time periods. This gap underscores the need to strengthen epidemiological monitoring at the regional level and to develop evidence-based preventive strategies.

Accordingly, the present study aims to provide a comprehensive assessment of the epidemiological characteristics of CAs registered in the Turkistan Region during the period from 2020 to 2024.

## 2. Materials and Methods

### 2.1. Study Design and Setting

This study was conducted as a retrospective observational epidemiological study analyzing cases of CAs registered in the Turkistan Region of Kazakhstan between 2020 and 2024.

**Figure 1:**
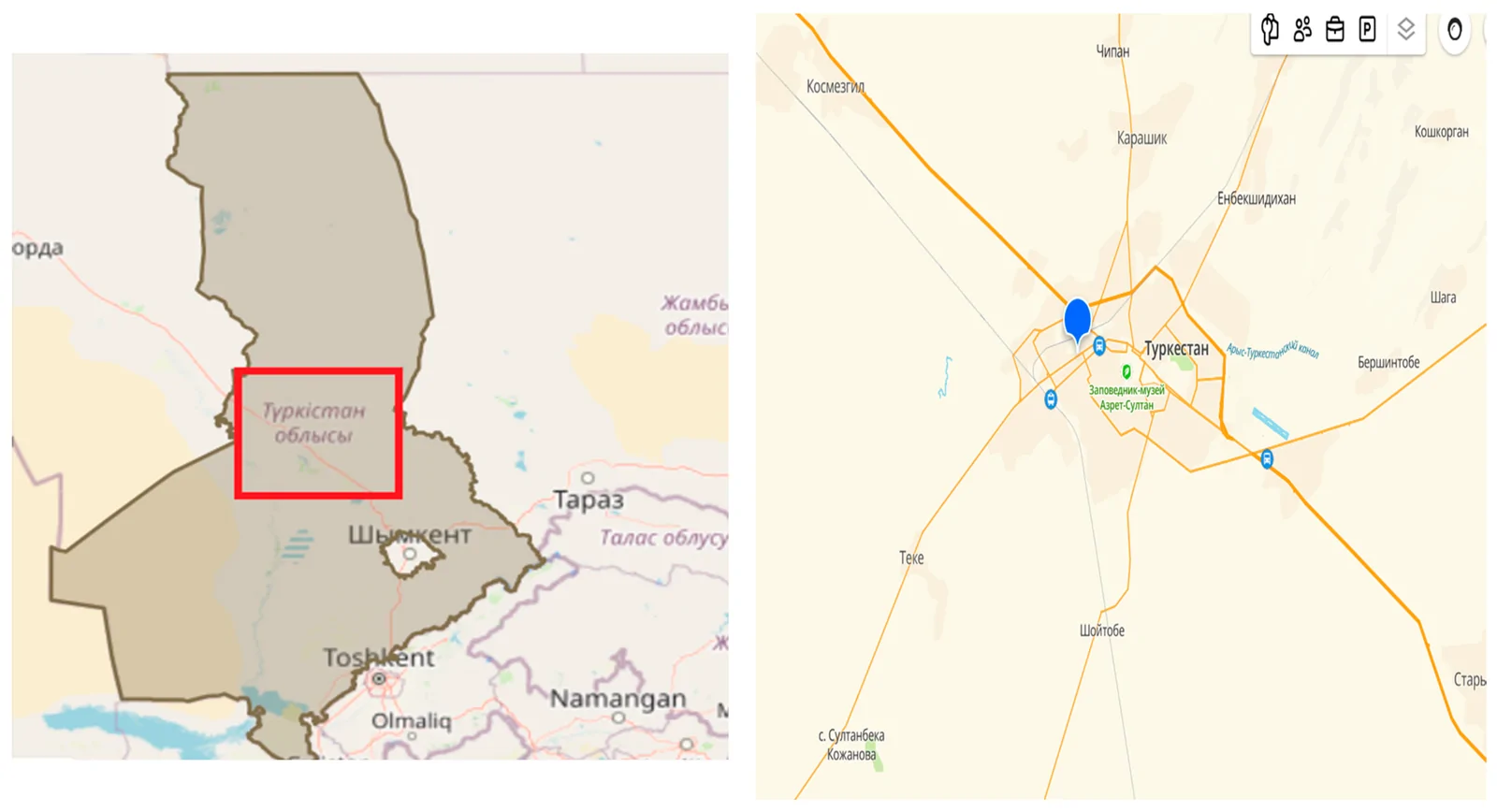
Study area and coverage of the Turkistan Regional Perinatal Center No. 3. Note: The label “Түркістан oблысы” in the figure refers to the Turkistan Region (Kazakhstan).

The study was carried out in the catchment area of the Turkistan Regional Perinatal Center No. 3, which provides specialized perinatal care for the cities of Turkistan and Kentau as well as the Otyrar, Sozak, and Sauran districts. The Turkistan Region is located in southern Kazakhstan and represents one of the most densely populated regions of the country. Kazakhstan is classified as an upper-middle-income country according to the World Bank classification [17]. The Turkistan Region is located in southern Kazakhstan and represents one of the most densely populated regions of the country. The study area, including the catchment population of the Turkistan Regional Perinatal Center No. 3 and affiliated districts, is shown in Figure 1.

The regional perinatal care system includes antenatal care services, prenatal screening programs, obstetric care, and neonatal assessment. Pregnant women undergo routine antenatal care in accordance with national clinical guidelines, including ultrasound examinations in the first and second trimesters and biochemical screening. Delivery care is primarily provided in healthcare facilities under the supervision of obstetricians and trained midwives [18]. Newborns undergo a routine clinical examination after birth, and suspected CAs are evaluated by neonatologists or pediatric specialists.

### 2.2. Study Population and Inclusion Criteria

The study population included all registered cases of CAs identified in healthcare institutions affiliated with the Turkistan Regional Perinatal Center No. 3 during the study period (2020–2024). The Turkistan Regional Perinatal Center No. 3 operates as an independent tertiary-level healthcare facility, representing the highest level of perinatal care in the regional healthcare system. Although the center does not have formally affiliated subsidiary institutions, it functions as a major referral and coordinating center for maternity care in the cities of Turkistan and Kentau, as well as the Otyrar, Sozak, and Sauran districts.

Within the framework of regionalized perinatal care, the center provides methodological and clinical oversight for obstetric services in the surrounding districts. On average, approximately 2% of all births in Kazakhstan (around every 50th newborn) occur at the Turkistan Regional Perinatal Center No. 3, indicating its substantial contribution to national perinatal care.

The analysis included:

- live-born infants diagnosed with congenital anomalies,
- stillbirths in which CAs were identified,
- cases of pregnancy termination due to prenatally diagnosed congenital anomalies.

CAs were classified according to the International Classification of Diseases, 10th Revision (ICD-10), codes Q00–Q99 [19]. The analysis focused primarily on major structural CAs diagnosed prenatally or at birth. Minor anomalies without clinical significance were not included unless they were associated with major congenital conditions.

### 2.3. Data Sources and Data Collection

Data were retrospectively collected from several sources within the regional perinatal healthcare system. These included:

- obstetric and gynecological medical records of pregnant women,
- delivery records and registration logs of the perinatal center,
- ultrasound examination reports and prenatal screening records,
- neonatal medical documentation,
- official statistical reports from regional healthcare institutions.

Data extraction was carried out by trained research personnel using a structured data extraction form specifically developed for this study. Information was obtained through review of both electronic medical records and archived paper-based documentation stored in the participating healthcare institutions.

The collected data were entered into a research database created for the study. Prior to analysis, all data were anonymized and personal identifiers were removed to ensure patient confidentiality. Only aggregated and de-identified data were used in the analysis.

### 2.4. Study Variables

The study variables included maternal, obstetric, neonatal, and socio-economic characteristics. A detailed description of the analyzed variables is presented in Table 1.

### 2.5. Case–Control Study of Risk Factors

A case–control study was conducted to assess potential risk factors associated with congenital anomalies. The study included 600 women from different districts of the Turkistan Region. The case group consisted of 300 women whose pregnancies were complicated by congenital anomalies or who delivered infants with congenital anomalies. The cases were selected using a consecutive sampling approach from all eligible congenital anomaly cases registered during the study period.

The control group included 300 women with physiologically normal pregnancies and healthy newborns. Controls were selected from the same healthcare institutions and during the same study period as the cases, using a random sampling approach. Women in the control group were selected from medical records of pregnancies without diagnosed congenital anomalies to ensure comparability with the case group.

Data on potential risk factors were collected using a structured questionnaire developed for this study. This questionnaire represented a specific component of the overall data collection process and was used to obtain detailed information on socio-demographic, medical, and behavioral factors not fully captured in routine medical records.

### 2.6. Statistical Analysis

Descriptive statistical methods were used to summarize the prevalence and distribution of congenital anomalies. Continuous variables were presented as means with standard deviations, while categorical variables were expressed as frequencies and percentages. Comparisons between groups were performed using chi-square tests for categorical variables. Associations between potential risk factors and CAs were evaluated using odds ratios (OR) with corresponding 95% confidence intervals. Prevalence rates and proportions were also calculated with corresponding 95% confidence intervals (95% CI).

A *p*-value of less than 0.05 was considered statistically significant. Statistical analyses were performed using IBM SPSS Statistics software 26.0 (IBM Corp., Armonk, NY, USA).

### 2.7. Ethical Considerations

The study protocol was reviewed and approved by the Ethics Committee of Khoja Akhmet Yassawi International Kazakh-Turkish University (Protocol No. 24, approved on 3 January 2024).

All data were anonymized prior to analysis and no personal identifiers were accessible to the research team. Due to the retrospective nature of the study and the use of de-identified data, the requirement for individual informed consent was waived by the ethics committee.

## 3. Results

During the study period (2020–2024), a total of 53,169 pregnancies were registered in the study area. Among these, 848 cases of CAs were identified, corresponding to an overall prevalence of 1.6%. Of these, 573 cases occurred among live births, 38 among stillbirths, and 237 were associated with pregnancy termination following prenatal diagnosis (95% CI: 1.49–1.71%).

The annual dynamics of congenital anomaly cases are presented in Table 1. In 2020, 79 cases were recorded (0.8% of pregnancies), increasing to 108 cases in 2021 (0.9%). A marked rise occurred in 2022, with 184 cases (1.7%), followed by 220 cases in 2023 (2.0%) and 257 cases in 2024 (2.4%). Overall, the prevalence increased from 0.8% (95% CI: 0.63–0.97%) in 2020 to 2.4% (95% CI: 2.11–2.69%) in 2024, representing a 3.2-fold increase.

The compound annual growth rate (CAGR) over the study period was approximately 34.3% per year. The largest increase was observed between 2021 and 2022 (70.4%), after which the growth rate slowed. The annual dynamics of congenital anomaly cases in the Turkistan Region during 2020–2024 are presented in Table 2.

**Figure 2:**
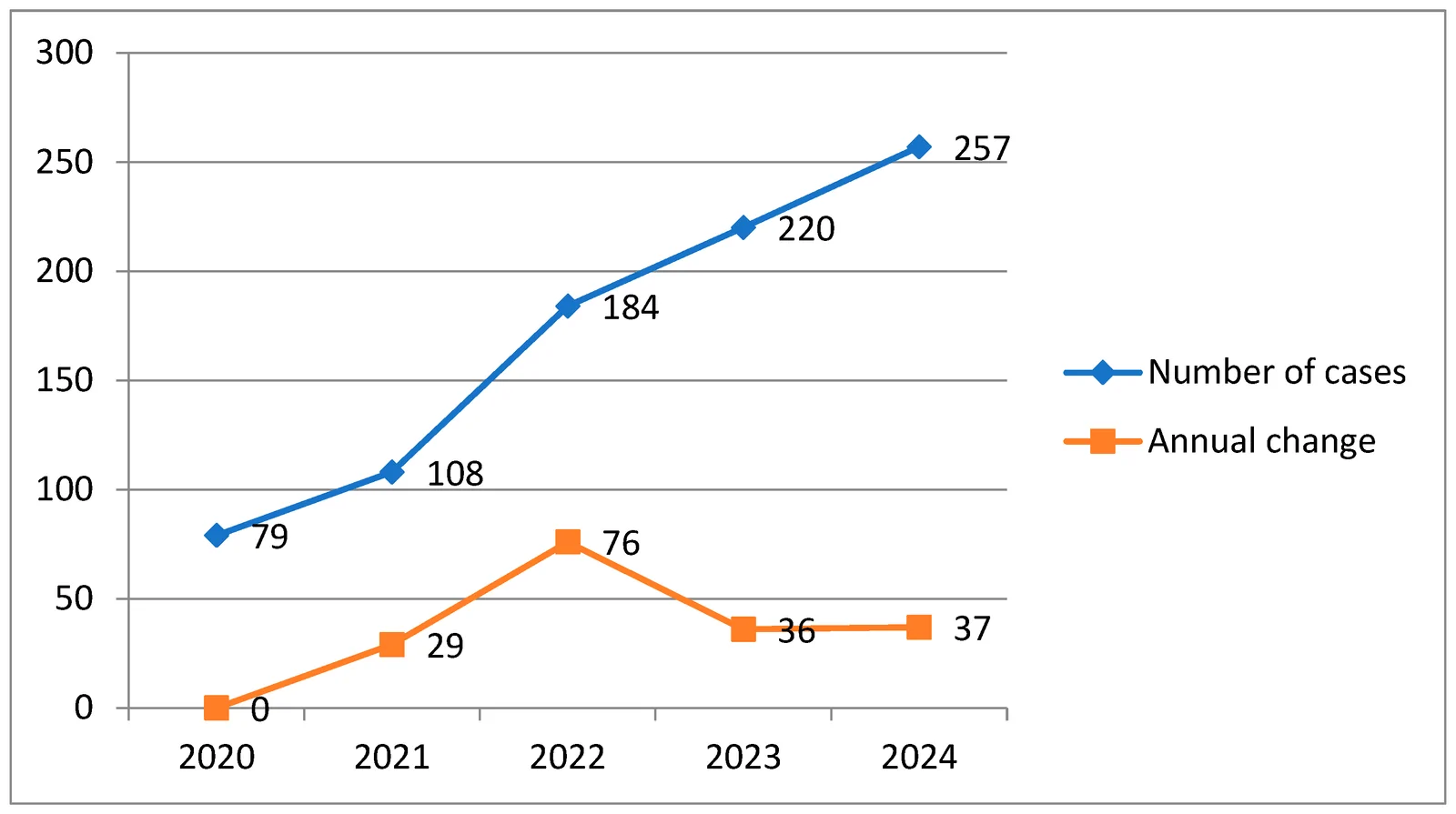
Annual number of CA cases and annual change (*n*), 2020–2024.

By 2024, the number of registered congenital anomaly cases was 225.3% higher than in 2020. Annual growth rates ranged from 16.8% to 70.4% during the study period, with the most pronounced increase occurring between 2021 and 2022 (Figure 2).

The distribution of CAs across organ systems during 2020–2024 is presented in Table 3.

Cardiovascular system anomalies (ICD-10 codes Q20–Q28) constituted the most frequently observed category of congenital anomalies. Their proportion increased from 22.2% in 2020 to 52.0% in 2024.

Digestive system anomalies accounted for 17.4% of cases in 2020 and 15.0% in 2023, but decreased to 6.0% in 2024.

Chromosomal disorders reached their highest proportion in 2023 (12.2%) and declined to 3.8% in 2024.

The proportion of cleft lip and palate ranged from 9% to 14% during 2020–2023 and increased to 16.3% in 2024.

Musculoskeletal anomalies accounted for 6.3% of cases in 2020, decreased to 4.0% in 2023, and increased to 9.8% in 2024.

**Figure 3:**
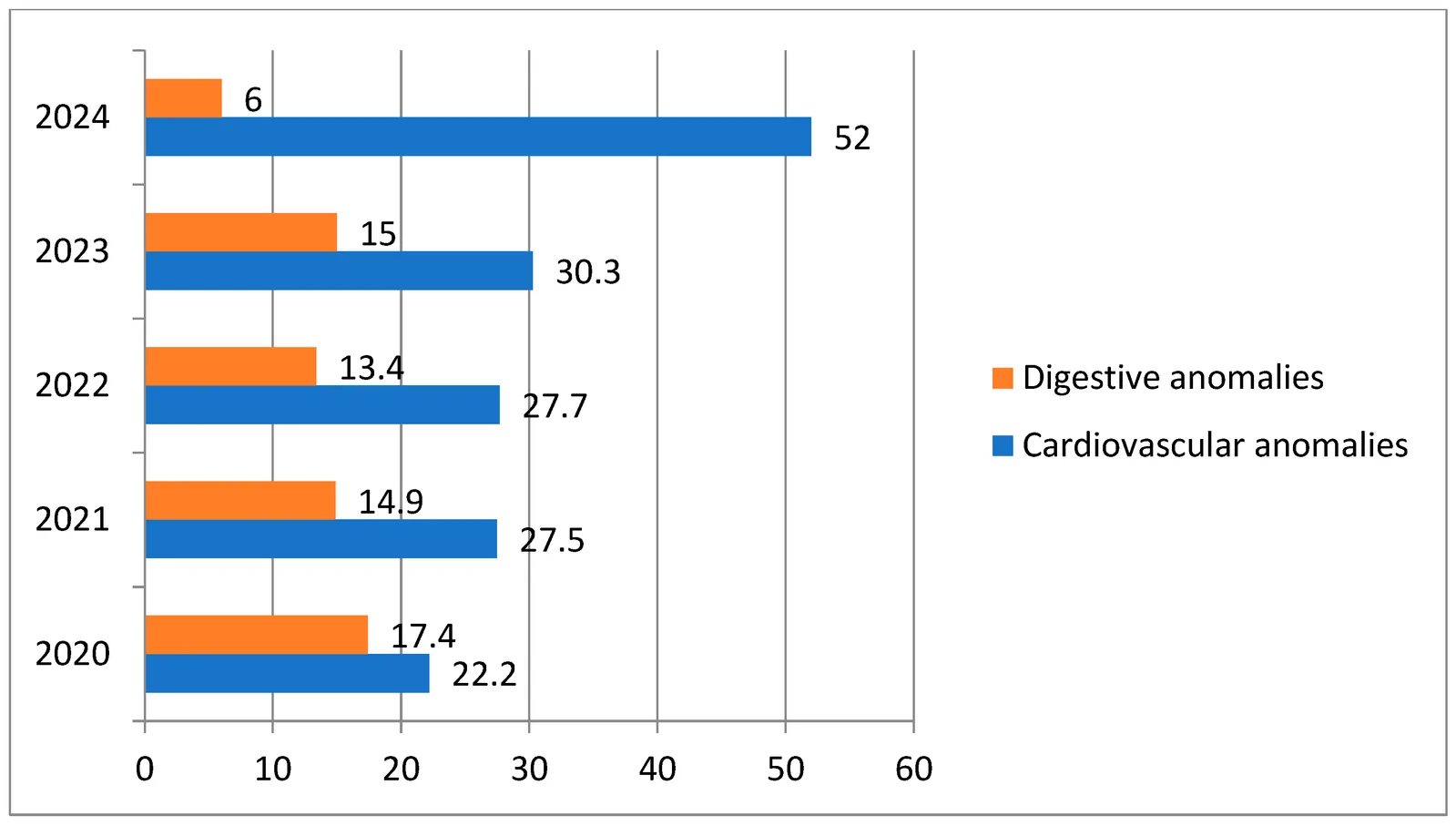
Temporal trends in the proportion (%) of cardiovascular and digestive system congenital anomalies, 2020–2024.

To illustrate changes in the structure of CAs over time, trends for two major anomaly groups were analyzed (Figure 3). Cardiovascular and digestive system anomalies were selected as they represented the largest proportions of cases during the study period.

The livebirth prevalence of congenital anomalies (CAs) in the Turkistan Regional Perinatal Center No. 3 was analyzed for the period 2020–2024 (Table 4). During this period, a total of 52,214 live births were recorded, including 573 infants diagnosed with congenital anomalies.

As shown in Table 4, the prevalence of CAs among live-born infants increased over the study period. The rate rose from 6.43 per 1000 live births (95% CI: 4.86–8.00) in 2020 to 17.96 per 1000 live births (95% CI: 15.40–20.52) in 2024. The total prevalence across the study period was approximately 11.0 cases per 1000 live births.

The prevalence of CAs among stillborn infants was also analyzed for the period 2020–2024 (Table 5).

During the study period, 489 stillbirths were registered, including 38 cases with congenital anomalies. The prevalence of CAs among stillborn infants varied across the study period, ranging from 26.09 to 113.64 cases per 1000 stillbirths.

### Terminations of Pregnancy (TOP)

During the study period (2020–2024), a total of 529 pregnancy terminations were registered in the Turkistan Regional Perinatal Center No. 3. Of these, 237 cases (44.8%) were associated with prenatally diagnosed congenital anomalies. The annual dynamics of pregnancy terminations associated with CAs are presented in Table 6. The number of such cases increased from 12 cases in 2020 to 90 cases in 2023, followed by a decrease to 65 cases in 2024.

The proportion of pregnancy terminations associated with CAs ranged from 33.96% to 57.32% during the study period, while the rate varied between 339.62 and 573.25 cases per 1000 pregnancy terminations.

**Figure 4:**
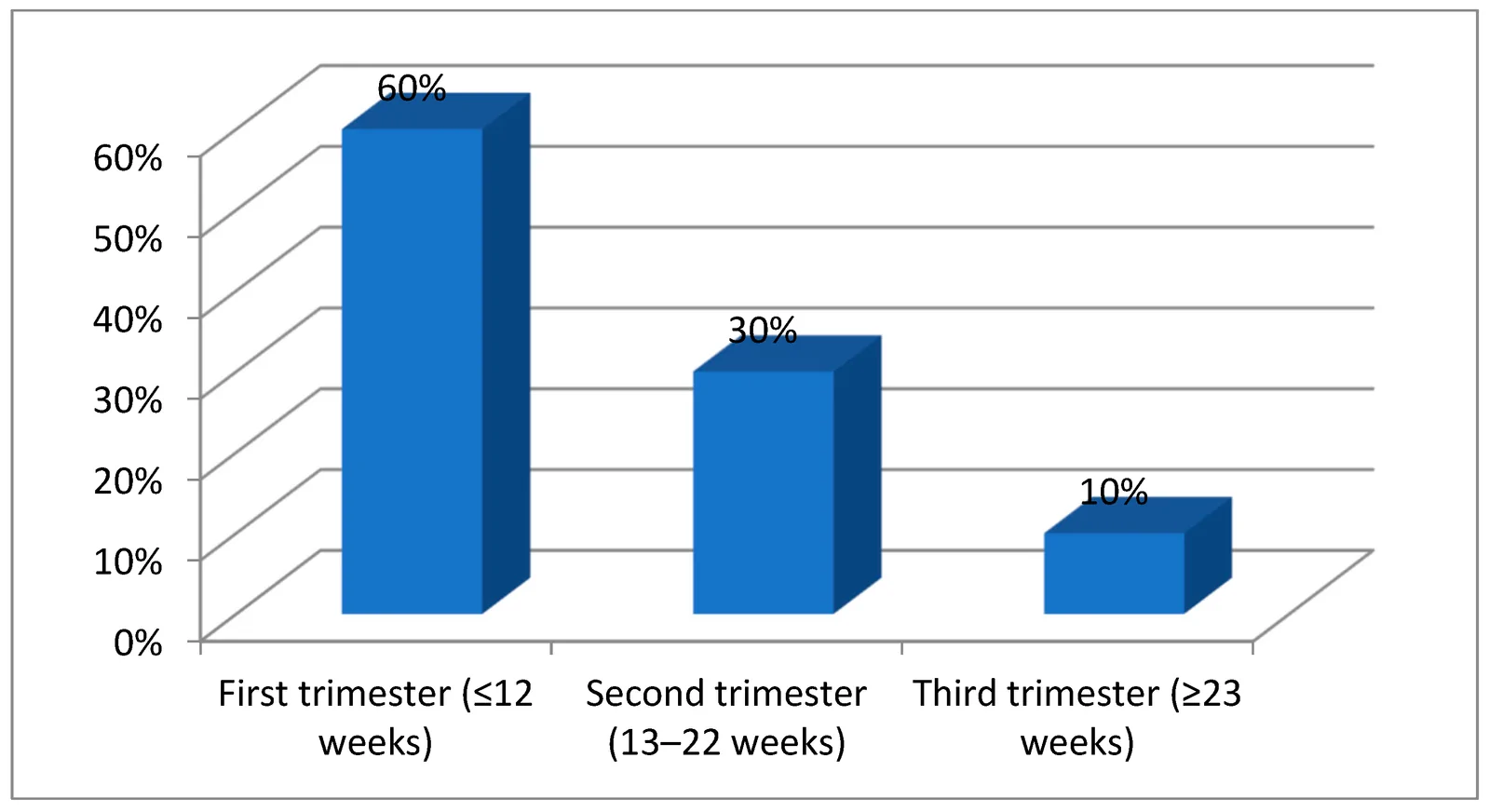
Distribution (%) of pregnancy terminations associated with congenital anomalies by gestational trimester in the Turkistan Region, 2020–2024.

Analysis by gestational age showed that most pregnancy terminations associated with CAs occurred in the first trimester, followed by the second trimester, whereas third-trimester terminations accounted for the smallest proportion. The distribution of pregnancy terminations associated with congenital anomalies by gestational trimester in the Turkistan Region during 2020–2024 is presented in Figure 4.

A comparative analysis of selected socio-demographic, medical, and behavioral risk factors was performed between the case and control groups. The distribution of these factors and their statistical significance are presented in Table 7.

Several factors were significantly more common in the case group, including adverse reproductive history, previous births with congenital anomalies, viral infections during pregnancy, medication exposure, and psychophysiological stress.

Viral infections during pregnancy were reported in 29.8% of women in the case group compared with 4.7% in the control group (*p* < 0.001).

## 4. Discussion

The aim of this study was to assess the prevalence, temporal trends, and potential determinants of CAs in the Turkistan Region between 2020 and 2024.

### 4.1. Prevalence of CAs in Regional and Global Context

The lower prevalence identified in the present study should be interpreted with caution. Differences between regional and international estimates may reflect variations in data sources, diagnostic capacity, and registration systems rather than true differences in the biological occurrence of congenital anomalies. In many low- and middle-income settings, CAs may be underreported due to limited access to prenatal diagnostic technologies, incomplete surveillance systems, or variations in clinical detection practices [20,21]. As diagnostic capacity expands, more cases may be identified that previously remained undetected, leading to an apparent increase in the recorded prevalence of CAs over time.

Continued strengthening of congenital anomaly registries and prenatal diagnostic services is therefore essential for improving the accuracy of epidemiological monitoring and guiding effective public health interventions.

### 4.2. Possible Explanations for Increasing Prevalence

The increasing prevalence of CAs observed in the present study may reflect a combination of improvements in diagnostic capacity, demographic changes, and environmental influences affecting maternal and fetal health [22].

One of the most plausible explanations for the observed trend in the Turkistan Region is the expansion of prenatal screening and diagnostic technologies during the study period. Advances in ultrasound imaging, biochemical screening, and genetic diagnostic methods have significantly improved the early detection of fetal abnormalities in many healthcare systems worldwide. As diagnostic capacity improves, cases that previously might have remained undetected or unregistered become increasingly identified and recorded in official health statistics [23]. The increasing number of pregnancy terminations associated with prenatally diagnosed CAs observed in this study may also indirectly reflect improved detection of severe fetal conditions during early pregnancy.

Improved awareness and clinical training among healthcare professionals may represent another contributing factor. Strengthening of perinatal healthcare services, increased attention to congenital anomaly surveillance, and better documentation practices can lead to more accurate identification and reporting of congenital anomalies. In many countries, the development of congenital anomaly registries and surveillance systems has been associated with an apparent rise in prevalence due to improved case ascertainment rather than a true increase in incidence [24].

The marked increase in registered congenital anomaly (CA) cases observed between 2020 and 2024 should be considered within the context of evolving prenatal screening and diagnostic practices in the Turkistan Region during the study period. Expansion of prenatal ultrasound screening, improved access to specialized diagnostic services, increased referral to perinatal centers, and enhanced clinical documentation may all have contributed to a greater likelihood of detecting and registering fetal anomalies. Consequently, the observed temporal increase in CA prevalence may reflect not only potential epidemiological changes, but also improvements in case identification, ascertainment, and reporting systems.

This interpretation is consistent with findings from previous epidemiological studies demonstrating that temporal increases in reported CA prevalence are frequently influenced by enhanced prenatal detection and surveillance practices rather than solely by true increases in underlying incidence. In regions undergoing improvements in maternal-fetal healthcare infrastructure, broader screening coverage and increased diagnostic sensitivity can substantially affect the number of identified cases.

Although environmental, demographic, and population-level factors may contribute to the overall burden of congenital anomalies, the relatively short time frame of the observed increase suggests that changes in screening coverage, diagnostic accessibility, and reporting practices likely played an important role in the trends identified in this study. Further prospective investigations incorporating environmental exposures, maternal risk factors, and standardized surveillance methodologies are needed to better distinguish true epidemiological changes from improvements in case detection and registration.

These findings highlight the importance of interpreting temporal trends in CA prevalence alongside concurrent developments in prenatal healthcare services and regional surveillance systems.

Demographic factors may also contribute to the observed epidemiological patterns. Increasing maternal age has been consistently identified as an important risk factor for several congenital anomalies, particularly chromosomal abnormalities. Global demographic trends toward delayed childbearing have therefore been associated with higher prevalence rates in many populations [25]. Although the present study did not primarily focus on demographic shifts, maternal age distribution may partly explain some of the observed regional patterns. Recent studies confirm that maternal age ≥35 years is associated with a significantly increased risk of chromosomal anomalies, including Down syndrome, due to age-related meiotic nondisjunction [26].

Environmental and lifestyle-related exposures may also play a role in shaping the epidemiology of congenital anomalies. Maternal infections, nutritional deficiencies, exposure to environmental pollutants, and certain medications during pregnancy have been identified as potential teratogenic factors affecting fetal development [27]. In regions undergoing rapid socioeconomic and environmental change, the interaction between these factors and healthcare system improvements may influence observed trends in congenital anomaly prevalence.

Taken together, the increasing prevalence observed in this study likely reflects a multifactorial process. In the context of the Turkistan Region, this trend may be attributed not only to changes in exposure to risk factors but also to improvements in prenatal diagnosis, case detection, and medical reporting systems. Understanding the relative contribution of these factors is essential for strengthening preventive strategies and optimizing maternal and child healthcare services aimed at reducing the burden of congenital anomalies.

### 4.3. Interpretation of Pregnancy Terminations (TOP)

The observed improvements in prenatal diagnosis are also reflected in patterns of pregnancy termination associated with congenital anomalies. Pregnancy termination associated with prenatally diagnosed CAs represents an important component of modern perinatal healthcare systems. In the present study, pregnancy termination was registered in 529 cases during the study period, of which 237 cases (44.8%) were associated with CAs detected during pregnancy. These findings indicate that prenatal diagnostic services are actively identifying fetal abnormalities and that clinical decisions regarding pregnancy management are being implemented within the regional healthcare system.

The distribution of pregnancy terminations by gestational age further supports the role of prenatal diagnostic services. In the present study, the majority of pregnancy terminations associated with CAs occurred during the first trimester of pregnancy [28]. Early detection of fetal abnormalities allows clinicians and families to make informed decisions regarding pregnancy management at earlier stages of gestation. Similar patterns have been reported in several international studies, where the expansion of prenatal screening programs has increased the proportion of CAs detected during early pregnancy [29].

The legal framework regulating pregnancy termination in Kazakhstan also plays an important role in shaping the observed patterns. According to the Order of the Minister of Health of the Republic of Kazakhstan No. ҚР ДСМ-122/2020 dated 9 October 2020, pregnancy termination may be performed upon the woman’s request up to 12 weeks of gestation, while termination for medical indications, including severe CAs of the fetus, may be performed at any stage of pregnancy if the condition is incompatible with life or associated with severe disability [30]. In addition, pregnancy termination for social indications is permitted up to 22 weeks of gestation.

This regulatory framework creates legal conditions for clinical decision-making when severe fetal abnormalities are detected during pregnancy. The relatively high proportion of pregnancy terminations associated with CAs observed in the present study may therefore reflect both the effectiveness of prenatal diagnostic services and the implementation of national clinical and legal regulations governing pregnancy termination.

At the same time, differences in access to prenatal diagnostic technologies, specialist consultations, and genetic counseling services may influence the timing and frequency of pregnancy terminations across regions. Strengthening early prenatal screening programs and improving access to specialized diagnostic services may further enhance the early detection of severe CAs and support informed reproductive decision-making.

### 4.4. Interpretation of Risk Factors

The findings of the present study demonstrate that the development of CAs is associated with a complex interaction between medical, genetic, behavioral, and socio-environmental factors. Several maternal and environmental characteristics were found to be significantly more common in the case group compared with the control group, indicating their potential contribution to the risk of congenital anomalies.

One of the most important associations identified in the study was the presence of an adverse reproductive history, including previous miscarriages, stillbirths, and previous births of children with congenital anomalies. One of the most important associations identified in the study was the presence of an adverse reproductive history, including previous miscarriages, stillbirths, and previous births of children with congenital anomalies. This is supported by previous studies, suggesting that both genetic susceptibility and persistent reproductive health conditions may contribute to repeated adverse pregnancy outcomes [31,32,33].

The study also demonstrated a strong association between infectious diseases during pregnancy and the occurrence of congenital anomalies. In particular, infections belonging to the TORCH group have long been recognized as important teratogenic factors capable of disrupting fetal development, particularly during early stages of embryogenesis. Viral infections such as toxoplasmosis, cytomegalovirus infection, and rubella have been shown to significantly increase the risk of structural and neurological abnormalities in the fetus.

Behavioral and lifestyle factors were also identified as important contributors to congenital anomaly risk. Maternal smoking, uncontrolled medication use during pregnancy, and increased psychophysiological stress were more frequently observed in the case group. Previous studies have demonstrated that exposure to harmful substances, teratogenic medications, and chronic stress during pregnancy may negatively affect fetal development and increase the risk of congenital anomalies [34,35,36].

In addition to medical and behavioral factors, the present study highlights the role of access to medical information and health literacy. Women in the case group were more likely to rely on informal sources of information, such as television and internet media, rather than professional medical consultations [37,38,39]. This finding suggests that insufficient medical literacy and limited access to reliable health information may reduce the effectiveness of preventive measures aimed at reducing the risk of congenital anomalies.

Taken together, the results of the present study indicate that CAs in the Turkistan Region are influenced by a combination of biological, environmental, and social factors.

### 4.5. Public Health Implications

Effective prevention strategies should therefore focus not only on clinical monitoring during pregnancy but also on strengthening preconception care, improving maternal health education, and expanding access to prenatal screening and genetic counseling services. The findings of the present study have several important implications for public health policy and the organization of perinatal care services in the Turkistan Region. The observed increase in the registered prevalence of congenital anomalies, together with the identification of multiple modifiable risk factors, highlights the need for strengthening preventive strategies aimed at reducing the burden of congenital anomalies.

First, the results emphasize the importance of improving preconception care and early pregnancy monitoring. Folate deficiency is a well-established risk factor for certain congenital anomalies, particularly neural tube defects and orofacial clefts. In Kazakhstan, national policies on folic acid supplementation and food fortification have been introduced prior to the study period, which may have contributed to the prevention of a proportion of these anomalies.

However, the extent to which mandatory folate fortification of staple foods is fully implemented, monitored, and enforced remains unclear. Partial coverage or variability in compliance may limit the effectiveness of this public health intervention. In addition, data on the actual population-level coverage of folate intake in the Turkistan Region are limited.

These factors may partially explain the persistence of preventable congenital anomalies observed in the present study and highlight the need for strengthened monitoring and evaluation of folate-related prevention programs. Many of the identified risk factors, including infectious diseases, harmful lifestyle behaviors, inadequate nutrition, and insufficient vitamin intake, may potentially be addressed through targeted preventive interventions before and during early pregnancy. International public health strategies increasingly emphasize the importance of preconception counseling and maternal health education as key components of congenital anomaly prevention programs.

Second, the study findings underline the critical role of antenatal screening and prenatal diagnostic services. Early identification of CAs allows timely referral to specialized medical centers, informed reproductive decision-making, and improved management of high-risk pregnancies. Strengthening access to high-quality prenatal screening programs, including ultrasound diagnostics and genetic counseling services, may significantly improve early detection of CAs and reduce adverse perinatal outcomes.

Third, the results highlight the importance of improving health literacy among women of reproductive age. The study demonstrated that women in the case group were more likely to rely on informal sources of health information rather than professional medical consultations. This finding suggests that expanding educational programs, strengthening communication between healthcare providers and patients, and promoting evidence-based health information may play an important role in reducing preventable risk factors associated with congenital anomalies.

In addition, strengthening epidemiological surveillance systems and congenital anomaly registries is essential for improving the accuracy of monitoring CAs at the regional and national levels. Reliable epidemiological data are necessary for identifying high-risk populations, evaluating the effectiveness of preventive programs, and guiding evidence-based public health policies.

### 4.6. Limitations of the Study

Despite the valuable findings obtained in this study, several limitations should be acknowledged. First, the study was based on a retrospective analysis of medical and statistical records from regional healthcare institutions. As a result, the accuracy of the collected data depended on the completeness and quality of the available medical documentation and reporting systems. Second, the study was conducted within a defined geographical area and included data primarily from healthcare institutions affiliated with the Turkistan Regional Perinatal Center No. 3. Therefore, the results may not fully represent the epidemiological situation in other regions of Kazakhstan, particularly in areas with different demographic characteristics, healthcare infrastructure, or environmental conditions.

Third, although a case–control design was used to assess potential risk factors associated with congenital anomalies, the analysis was limited to selected variables collected through medical records and structured questionnaires. Some potentially important factors, including detailed environmental exposures, genetic determinants, and long-term maternal health indicators, were not available in the dataset and therefore could not be included in the analysis.

In addition, potential sources of bias should be considered when interpreting the findings of this study. The use of multiple data sources, including medical records, statistical reports, and questionnaire-based data collection, may introduce information bias due to differences in data quality and completeness. Furthermore, the case–control component relied partly on self-reported information, which may be subject to recall bias. These factors could influence the observed associations and should be taken into account when interpreting the results.

Furthermore, the identification of CAs depended on diagnostic practices and the availability of prenatal and neonatal diagnostic technologies. Improvements in diagnostic capacity over time may have contributed to the increasing number of detected cases, which could partially influence the observed temporal trends.

In addition, changes in prenatal screening coverage, referral pathways, clinical awareness, and case registration practices during the study period may have influenced the comparability of prevalence estimates across years. Because detailed annual data regarding the implementation and coverage of screening programs were not available, it was not possible to distinguish between true epidemiological changes and improvements in case detection and ascertainment. Therefore, the observed increase in registered CA prevalence should be interpreted within the context of evolving diagnostic and surveillance systems in the region.

Finally, the retrospective design of the study does not allow for the establishment of causal relationships between the identified risk factors and the occurrence of congenital anomalies. The associations observed in this study should therefore be interpreted with caution and require further investigation in prospective and multicenter studies.

### 4.7. Recommendations for Research and Policy

Further research should focus on strengthening epidemiological monitoring of congenital anomalies through multicenter and prospective studies across different regions of Kazakhstan. In particular, additional investigation of environmental exposures, genetic factors, and maternal health conditions is needed to better understand the determinants of congenital anomalies. Evaluating the effectiveness of prenatal screening programs and preventive interventions is also essential for improving early detection and prevention strategies.

Future studies should also aim to distinguish temporal changes related to improved case detection and surveillance practices from true epidemiological changes in congenital anomaly prevalence.

From a public health perspective, priority actions should include expanding preconception counseling services, improving access to prenatal screening and genetic counseling, and strengthening educational programs to enhance health literacy among women of reproductive age. In addition, the development of comprehensive regional surveillance systems would improve the accuracy of epidemiological monitoring and support evidence-based decision-making in maternal and child healthcare.

## 5. Conclusions

This study identified an increase in the registered prevalence of congenital anomalies in the Turkistan Region between 2020 and 2024. The observed temporal trend likely reflects a combination of factors, including improvements in prenatal screening, diagnostic capacity, case ascertainment, and reporting practices, together with possible epidemiological influences. The findings highlight the importance of strengthening congenital anomaly surveillance systems, expanding access to high-quality prenatal diagnostic services, and improving preventive maternal healthcare programs. Further prospective and multicenter studies are needed to better distinguish true epidemiological changes from evolving detection and registration practices.

## Figures and Tables

**Table 1:** Study variables included in the analysis.

Category	Variables
Maternal characteristics	Maternal age; education level; place of residence (urban/rural); occupational exposure; chronic diseases; infectious diseases during pregnancy
Pregnancy and obstetric history	Previous pregnancies; miscarriages; stillbirths; preterm deliveries; previous births of children with congenital anomalies
Prenatal diagnostic indicators	Participation in first- and second-trimester screening; ultrasound findings; genetic counseling; invasive diagnostic procedures (amniocentesis, chorionic villus biopsy, cordocentesis)
Neonatal characteristics	Birth weight; birth length; Apgar score; viability status; anatomical type of congenital anomaly
Environmental and socio-economic factors	Maternal lifestyle characteristics; dietary patterns; environmental exposures; access to healthcare services

**Table 2:** Annual dynamics of congenital anomaly cases in the Turkistan Region, 2020–2024.

Year	Cases (*n*)	Annual Change (*n*)	Annual % Change	Index (2020 = 100)
2020	79	–	–	100
2021	108	29	36.7	136.7
2022	184	76	70.4	232.9
2023	220	36	19.6	278.5
2024	257	37	16.8	325.3
Total	848			

**Table 3:** Distribution of congenital anomalies by organ system, 2020–2024 (%).

Category of Congenital Anomaly	2020	2021	2022	2023	2024
Cardiovascular system	14 (22.2%)	24 (27.5%)	33 (27.7%)	37 (30.3%)	94 (52.0%)
Digestive system	11 (17.4%)	13 (14.9%)	16 (13.4%)	18 (15.0%)	11 (6.0%)
Cleft lip and palate	9 (14.2%)	12 (13.8%)	14 (11.7%)	11 (9.0%)	20 (16.3%)
Musculoskeletal system	4 (6.3%)	5 (5.7%)	6 (5.0%)	5 (4.0%)	12 (9.8%)
Central nervous system	4 (6.3%)	6 (6.8%)	11 (9.2%)	15 (12.2%)	7 (3.8%)
Urinary system	3 (4.7%)	4 (4.6%)	6 (5.0%)	7 (5.7%)	7 (3.8%)
Respiratory system	3 (4.7%)	3 (3.4%)	6 (5%)	5 (4.09%)	5 (2.7%)
Chromosomal disorders	4 (6.3%)	6 (6.8%)	11 (9.2%)	15 (12.2%)	7 (3.8%)

**Table 4:** Congenital anomalies among live-born infants, 2020–2024.

Year	Total Livebirths	Livebirths with CAs (*n*)	Rate per 1000 Live Births
2020	9793	63	6.43
2021	11,413	87	7.62
2022	10,518	119	11.31
2023	10,355	122	11.78
2024	10,135	182	17.96
Total	52,214	573	11.0

**Table 5:** Congenital anomalies among stillborn infants, 2020–2024.

Year	Total Stillbirths	Stillbirths with CAs (*n*)	Rate per 1000 Stillbirths
2020	88	4	45.45
2021	115	3	26.09
2022	117	13	111.11
2023	81	8	98.77
2024	88	10	113.64
Total	489	38	77.7

**Table 6:** Annual dynamics of pregnancy terminations associated with congenital anomalies in the Turkistan Regional Perinatal Center No. 3, 2020–2024.

Year	Total Pregnancy Terminations	Terminations Associated with Congenital Anomalies	Proportion (%)	Rate per 1000 Pregnancy Terminations
2020	35	12	34.29	342.86
2021	53	18	33.96	339.62
2022	131	52	39.69	396.95
2023	157	90	57.32	573.25
2024	153	65	42.48	424.83
Total	529	237	44.8	448.0

**Table 7:** Comparison of selected risk factors between case and control groups.

Risk Factor	Case Group (*n* = 300, %)	Control Group (*n* = 300, %)	*p*-Value
Chronic diseases during pregnancy	54.6	14.7	<0.001
Adverse reproductive history	43.7	31.0	0.001
Psychophysiological stress	41.4	5.7	<0.001
Irregular diet	36.6	12.0	<0.001
Physical workload	35.4	8.3	<0.001
Medication use during pregnancy	30.1	2.0	<0.001
TORCH infections	29.8	4.7	<0.001
Viral infections	20.9	3.0	<0.001
Hereditary diseases in paternal relatives	18.9	0.3	<0.001
Previous child with CA	11.6	0.7	<0.001
Infrequent fruit/vegetable intake	11.0	2.7	0.001
Hereditary diseases in maternal relatives	10.9	0	<0.001
Maternal smoking	7.3	0.7	<0.001

## Data Availability

The original contributions presented in this study are included in the article. Further inquiries can be directed to the corresponding author.

## Abbreviations

The following abbreviations are used in this manuscript:

CAs	Congenital anomalies
CHDs	Congenital heart defects
VSD	Ventricular septal defect
PDA	Patent ductus arteriosus
